# Expression of zinc finger transcription factors (ZNF143 and ZNF281) in serous borderline ovarian tumors and low-grade ovarian cancers

**DOI:** 10.1186/s13048-019-0501-9

**Published:** 2019-03-18

**Authors:** Paweł Sadłecki, Marek Grabiec, Dariusz Grzanka, Jakub Jóźwicki, Paulina Antosik, Małgorzata Walentowicz-Sadłecka

**Affiliations:** 1 Department of Obstetrics and Gynecology, Collegium Medicum in Bydgoszcz, Nicolaus Copernicus University in Torun, ul. Ujejskiego 75, 85-168 Bydgoszcz, Poland; 2 Department of Clinical Pathomorphology, Collegium Medicum in Bydgoszcz, Nicolaus Copernicus University in Torun, Bydgoszcz, Poland

**Keywords:** Low-grade ovarian cancer, Ovarian cancer, Borderline ovarian tumor, Epithelial-mesenchymal transition, Transcription factors, ZNF143, ZNF281

## Abstract

Low-grade ovarian cancers represent up to 8% of all epithelial ovarian carcinomas (EOCs). Recent studies demonstrated that epithelial-mesenchymal transition (EMT) is crucial for the progression of EOCs. EMT plays a key role in cancer invasion, metastasis formation and chemotherapy resistance. An array of novel EMT transcription factors from the zinc finger protein family have been described recently, among them zinc finger protein 143 (ZNF143) and zinc finger protein 281 (ZNF281). The study included tissue specimens from 42 patients. Based on histopathological examination of surgical specimens, eight lesions were classified as serous borderline ovarian tumors (sBOTs) and 34 as low-grade EOCs. The proportions of the ovarian tumors that tested positively for ZNF143 and ZNF281 were 90 and 57%, respectively. No statistically significant differences were found in the expressions of ZNF143 and ZNF281 transcription factors in SBOTs and low-grade EOCs. Considering the expression patterns for ZNF143 and ZNF281 identified in this study, both sBOTs and low-grade EOCs might undergo a dynamic epithelial-mesenchymal interconversion. The lack of statistically significant differences in the expressions of the zinc finger proteins in sBOTs and low-grade serous EOCs might constitute an evidence for common origin of these two tumor types.

## Introduction

Epithelial ovarian cancer (EOC) is the eighth most common female cancer worldwide; EOC accounts for up to one-fourth of all female genital malignancies and is associated with the highest mortality of all genital cancers [1]. The classification system for EOCs was modified in the last decade to be consistent with the novel concept of a two-tier grading system in which serous cancers are divided into high- and low-grade malignancies [2]. Low-grade or type I ovarian cancers represent 6–8% of all EOCs. They are usually well-differentiated and have variable histological structure; this group includes low-grade endometrioid, clear-cell, mucinous and low-grade serous ovarian carcinomas. Borderline tumors constitute approximately 10–20% of all epithelial ovarian lesions [3]. Low-grade ovarian cancers are characterized by slow growth, approximately a 55% five-year survival rate and high resistance to chemotherapy. On the first pathway, normal ovarian tissues undergo transformation into a borderline tumor, which may later progress to low-grade serous carcinoma (LGSC), mucinous, endometroid or clear-cell tumor [4]. The majority of women with EOCs are treated with primary debulking surgery and chemotherapy. Standard combination chemotherapy includes a platinum agent and taxane [5]. When low-grade cancers are confined to the ovary, the prognosis is good; however, due to resistance to chemotherapy and recurrence of the disease, long-term survival in advanced stages remains poor. Low- and high-grade serous ovarian cancers are now considered separate tumor entities with different clinical and molecular characteristics [4, 6]. Most low-grade tumors are genetically stable, display a wild-type (wt) TP53 and show alterations in PIK3CA, PTEN, KRAS, ERK and ARID1A genes [7].

Epithelial-mesenchymal transition (EMT) is a reversible process during which epithelial cells lose their characteristic features, such as polarity and potential for cell-to-cell interactions [8]. In ovarian malignancies, EMT contributes to greater mobility and invasiveness, and boosts up metastatic potential of tumor cells which gain typical characteristics of cancer stem cells [9]. Moreover, the EMT phenotype was shown to be associated with drug resistance, and hence, may predispose to recurrence and metastasis after a standard chemotherapy [10, 11]. A number of classical transcription factors, such as SNAIL, SLUG, TWIST and ZEB, play important roles in the regulation of EMT, contributing to a decrease in E-cadherin expression [8]. Moreover, an array of novel EMT transcription factors from the zinc finger protein family have been described recently, among them zinc finger protein 143 (ZNF143) and zinc finger protein 281 (ZNF281).

ZNF143 is a cell cycle-related transcription factor, first described as a human homolog of the Xenopus selenocysteine tRNA gene transcription activating factor (Staf) [12]. ZNF143 was shown to modulate cell survival, controlling activity of glutathione peroxidase 1 through the transcriptional activation of selenocysteine transfer RNA [13]. Moreover, this protein was postulated to regulate the replication of DNA and cell cycle-associated genes involved in cell growth and proliferation [14]. To this date, the function of ZNF143 has been studied in various malignancies, including leukemia, lung adenocarcinoma, colon cancer, prostate cancer, gastric cancer and breast cancer [14–17].

ZNF281 is an EMT-promoting transcription factor; it plays a similar role as the structurally-related transcription factors: SNAIL, SLUG and ZEB1/2 [18]. The first evidence for a presumable involvement of ZNF281 in EMT was the observation that this protein is regulated by SOX4, an EMT-inducing factor [19]. SOX4 is critical for vertebrate development, as it coordinates differentiation and proliferation of cells in various tissues. Moreover, SOX4 was shown to induce the transcription of ZNF281 directly [19]. This transcription factor has also been implicated in the regulation of EMT, and its overexpression has already been found in many human malignancies [20, 21]. The fact that enhanced expression of ZNF281 in colorectal cancer was shown to correlate significantly with the tumor stage, implies that this protein might be useful in the diagnostics of other human malignancies, establishing prognosis and perhaps also anticancer therapy [22].

However, still little is known about the role of ZNF143 and ZNF281 in the pathogenesis of ovarian neoplasms, and published evidence in this matter is sparse and inconclusive. The aim of this study was to analyze the expressions of ZNF143 and ZNF281 in tumor tissues and to verify if they correlate with clinicopathological characteristics of borderline ovarian tumors and low-grade ovarian cancers. Moreover, we compared the expressions of ZNF143 and ZNF281 in these two groups of ovarian tumors.

## Methods

A total of 42 patients diagnosed with ovarian tumors and treated at the Department of Obstetrics and Gynecology, Nicolaus Copernicus University, Collegium Medicum in Bydgoszcz (Poland), were enrolled to the study. The study included patients whose ovarian tumors were classified as serous borderline ovarian tumors (sBOTs, *n* = 8) or low-grade ovarian cancers (*n* = 34) based on histopathological examination of surgical specimens. Patients with benign tumors of the ovaries, high-grade type 2 ovarian carcinomas and metastatic ovarian tumors were excluded from the study. Between January 2009 and June 2012, all patients underwent clinical stage-appropriate surgical resections of ovarian tumors, and whenever necessary, received adjuvant platinum-based chemotherapy as recommended by current Polish guidelines [23]. Clinicopathological characteristics of the study patients are summarized in Table 1. The study design, data analysis and interpretation, drafting and revisions of the manuscript followed the Strengthening the Reporting of Observational Studies in Epidemiology (STROBE) Statement: guidelines for reporting observational studies, available through the EQUATOR (enhancing the quality and transparency of health research) network (http://www.equator-network.org/).

**Table 1:** Clinicopathological characteristics of the study participants

	N	%
Age (years)		
≤ 50	12	28.5%
> 50	30	71.5%
Histological type		
Serous	13	30.9%
Mucinous	3	7.2%
Endometrioid	8	19.0%
Clear cell	10	23.9%
Serous borderline tumor	8	19.0%
Figo (stage)		
I A	29	69.0%
I B	5	11.9%
I C	5	11.9%
Other	3	7.2%
Grade		
-	8	19.0%
G 1	7	16.7%
G 2	27	64.3%

### Tissue macroarrays

Immunohistochemical analysis of formalin-fixed, paraffin-embedded (FFPE) tissue specimens was carried out at the Department of Clinical Pathology, Nicolaus Copernicus University, Collegium Medicum in Bydgoszcz. Before preparation of tissue macroarrays, original hematoxylin and eosin-stained microscopic slides were analyzed by two independent pathologists to identify the most representative tissue areas. Then, these areas were cut out from five primary FFPE tissue fragments and transferred to a donor block, creating a tissue macroarray. Using a rotary microtome (Accu-Cut®SMRTM200, Sakura, Japan), the paraffin-embedded donor blocks were cut into 3-μm-thick slices which were then used for immunohistochemical studies.

### Immunohistochemistry

Automated immunostaining for ZNF143 and ZNF281 was carried out with EnVision FLEX+ HRP reagents (Dako, Agilent Technologies, INC., Santa Clara, CA, USA). The immunostaining protocol included: (a) deparaffinization, rehydration and antigen retrieval with Epitope Retrieval Solution High-pH (Dako, Agilent Technologies, Santa Clara, CA, USA) in PT Link (Dako, Agilent Technologies), followed by rinsing with wash buffer, (b) 10-min treatment with 3% hydrogen peroxide to block the activity of endogenous peroxidase, followed by rinsing with wash buffer, (c) 15-min incubation with 3% bovine serum albumin, followed by rinsing with wash buffer, (d) 30-min incubation with mouse monoclonal anti-ZNF143 antibody (ab58168, Abcam, Cambridge, UK) and rabbit polyclonal anti-ZNF281 antibody, diluted 1:100 and 1:50 with Dako Antibody Diluent (Dako, Agilent Technologies, Inc., Santa Clara, CA, USA), (e) 2 × 5-min rinsing with wash buffer, (f) 15-min incubation with EnVision Flex+ Rabbit/Mouse (LINKER) to enhance the reaction, followed by 2 × 5-min rinsing with wash buffer, (g) 20-min incubation in EnVision FLEX+ HRP (Dako, Agilent Technologies, Inc., Santa Clara, CA, USA), (h) 5-min incubation in 3,3′-diaminobenzidine (DAB) solution, (i) rinsing with water, (j) rinsing with wash buffer, and (k) 5-min counterstaining with hematoxylin. The same steps were repeated for human ovary (clear cell carcinoma) and human cerebellum (Purkinje cells) tissues, used as positive controls as recommended in the antibody datasheet and the Human Protein Atlas [24]. During preparation of negative controls, 1% BSA solution in PBS was used instead of the primary antibodies. All procedures were carried out at room temperature. Finally, the slides were dehydrated in alcohol gradient, cleared in xylene and sealed with Dako mounting medium (Agilent Technologies, Inc. Santa Clara, CA, USA).

### Evaluation of immunohistochemical reactions

The antibody-labeled slides were evaluated by two independent pathologists under a low-power (× 20) ECLIPSE E800 light microscope (Nikon Instruments Europe, Amsterdam, Netherlands). The immunoexpression of the analyzed proteins in ovarian tissue macroarrays was quantified using Remmele-Stegner (IRS) scoring system. The IRS score for each macroarray spot was calculated by multiplying staining intensity (0 = negative, 1 = weakly positive, 2 = moderately positive, 3 = strongly positive) by the proportion of positively stained cells (1 = 1–9%, 2 = 10–50%, 3 = 51–80%, 4 = 81–100%); hence, the final scores might vary between 0 and 12. Statistical analysis included mean IRS score for all tissue macroarrays [25].

### Statistical analysis

Statistical analysis was carried out with PQStat package, version 1.6.4.121. The significance of intergroup differences was verified with Mann-Whitney U-test (k = 2) or Kruskal-Wallis test (k > 2). Survival curves were compared with log-rank test, Wilcoxon-Breslow-Gehan test and Taron-Ware test. The results were considered significant at *p* < 0.05 and highly significant at *p* < 0.01.

### Ethics

The protocol of the study was approved by the Local Bioethics Committee at the Nicolaus Copernicus University, Collegium Medicum in Bydgoszcz (decision no. KB 413/2016), and written informed consent was sought from each patient or her next of kin.

## Results

**Fig. 1:**
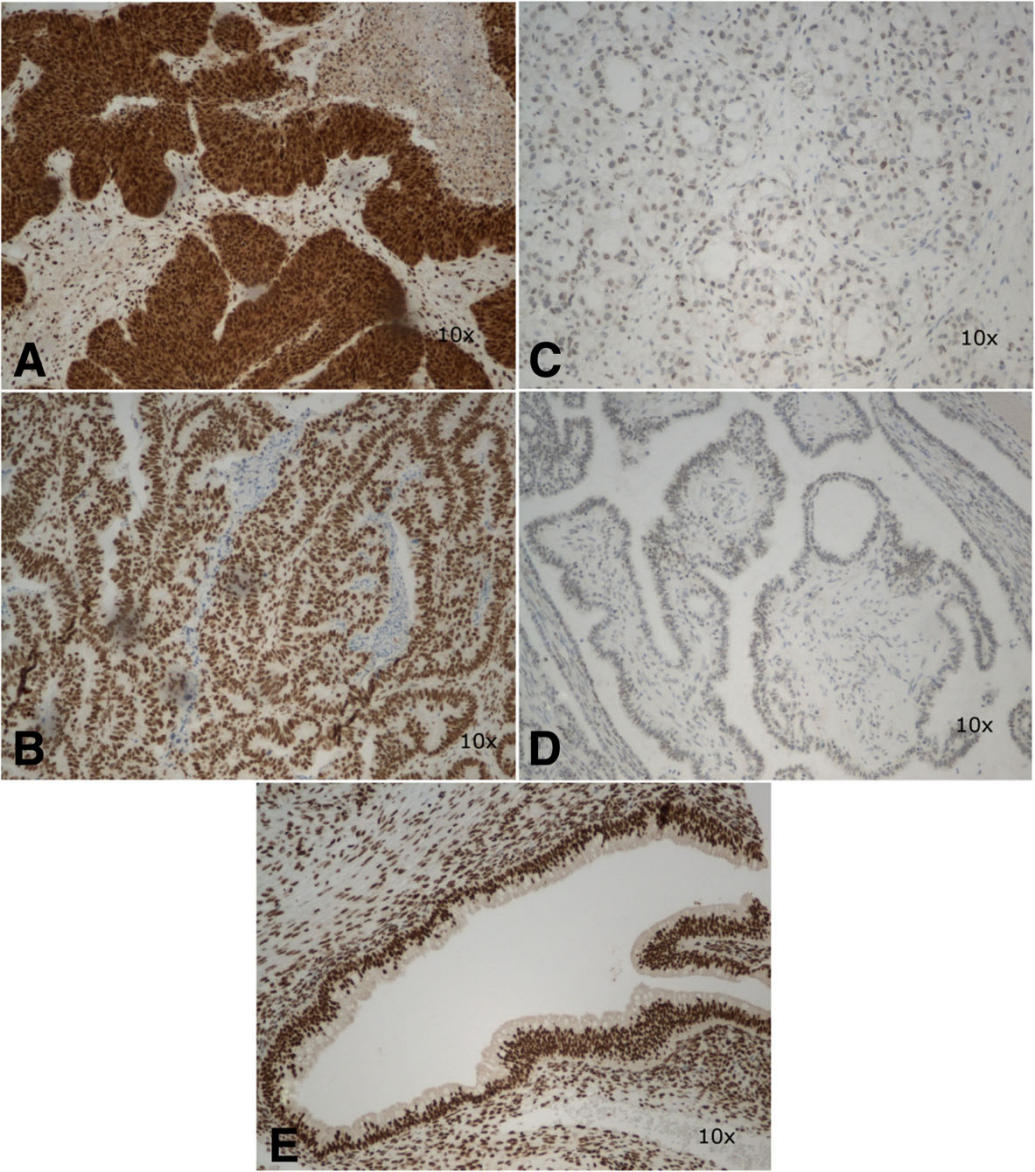
Microphotograph presenting strong nuclear expression of ZNF143 in clear-cell ovarian carcinoma (**a**) and borderline tumor (**b**), weak nuclear expression in endometrioid ovarian cancer (**c**) and borderline tumor (**d**), and positive expression in ovarian stroma and epithelium of normal phenotype (**e**). Magnification × 10

**Fig. 2:**
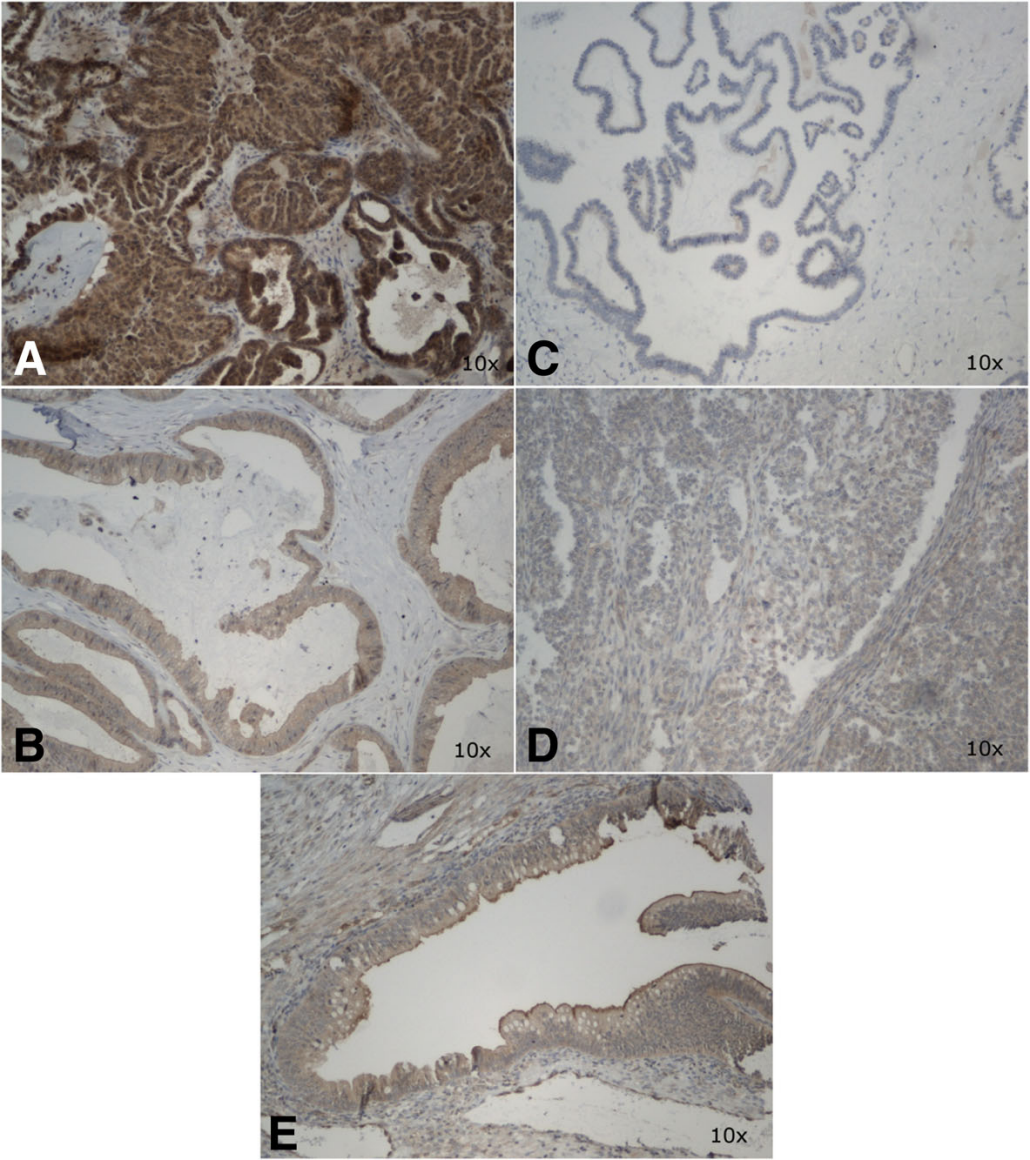
Microphotograph presenting cytoplasmic expression of ZNF281 in endometrioid ovarian cancer (**a**) and borderline tumor (**b**), negative staining in clear-cell ovarian cancer (**c**) and borderline tumor (**d**), and weak expression in ovarian stroma and epithelium of normal phenotype (**e**). Magnification × 10

The expression of ZNF 143 was found in 90% (*N* = 38) of examined specimens; in all cases (*N* = 38), the expression was classified as strong. The expression of ZNF 281 was observed in 57% (*N* = 24) of the specimens, including 25% of the specimens (*N* = 6) in the case of which it was classified as strong. While the expression of ZNF 143 was found primarily in cell nuclei, ZNF 281 was expressed in the cytoplasm. No statistically significant differences were found in the expressions of ZNF143 and ZNF281 in borderline tumors and low-grade ovarian cancers (Table 2). Representative microphotographs presenting expressions of various transcription factors in the analyzed tissues are shown in Figs. 1 and 2. The expressions of ZNF143 and ZNF 281 did not differ significantly depending on clinicopathological characteristics of the ovarian tumors: histopathological type, clinical stage (1A vs. others) and histological grade (G1 vs. G2). Moreover, no statistically significant differences in the expressions of the transcription factors were found when menopausal status of the patients was considered as a grouping variable (Tables 3 and 4). While none of the patients with sBOTs died during a 5-year follow-up period and four deaths were documented among women with low-grade ovarian cancers, no significant between-group differences in the survivals were found on statistical analysis.

**Table 2:** Immunoexpression of the EMT transcription factors stratified according to the type of ovarian tumor (sBOT – serous borderline ovarian tumor, EOC – epithelial ovarian cancer)

Group	Arithmetic mean	Standard deviation	Minimum	Lower quartile	Median	Upper quartile	Maximum	Mann-Whitney U-test
ZNF 281								
sBOT	3.13	2.36	0.00	2.00	2.50	4.00	8.00	Z = 1.1260 *p* = 0.2602
EOC	3.79	2.14	0.00	3.00	4.00	4.00	8.00	
ZNF 143								
sBOT	10.00	4.28	0.00	11.00	12.00	12.00	12.00	Z = 0.0208 *p* = 0.9834
EOC	10.56	2.82	0.00	9.75	12.00	12.00	12.00

**Table 3:** Immunoexpression of the ZNF 143, stratified according to histopathological type, clinical stage, histological grade of ovarian tumors and menopausal status of the study patients (“other” corresponds to stage IB and higher clinical stages of ovarian tumors)

ZNF 143	Group	Arithmetic mean	Standard deviation	Minimum	Lower quartile	Median	Upper quartile	Maximum	Mann-Whitney U-test
Histopathological type	Borderline	10.00	4.28	0.00	11.00	12.00	12.00	12.00	H = 1.7106 *p* = 0.7888
	Clarocellulare	10.20	3.05	4.00	9.00	12.00	12.00	12.00	
	Endometrioidales	9.63	4.21	0.00	8.75	12.00	12.00	12.00	
	Mucinosum	12.00	0.00	12.00	12.00	12.00	12.00	12.00	
	Serosum	11.08	1.75	8.00	12.00	12.00	12.00	12.00	
Stage	IA	10.41	2.99	0.00	8.00	12.00	12.00	12.00	Z = 0.3527 *p* = 0.7243
	Other	10.54	3.43	0.00	12.00	12.00	12.00	12.00	
Grade	G1	10.50	2.07	8.00	8.00	12.00	12.00	12.00	Z = 0.6029 *p* = 0.5466
	G2	10.58	3.05	0.00	12.00	12.00	12.00	12.00	
Menopausal status	Postmenopausal	10.41	2.99	0.00	8.00	12.00	12.00	12.00	Z = 0.3527 *p* = 0.7243
	Premenopausal	10.54	3.43	0.00	12.00	12.00	12.00	12.00

**Table 4:** Immunoexpression of the ZNF 281, stratified according to histopathological type, clinical stage, histological grade of ovarian tumors and menopausal status of the study patients (“other” corresponds to stage IB and higher clinical stages of ovarian tumors)

ZNF 281	Group	Arithmetic mean	Standard deviation	Minimum	Lower quartile	Median	Upper quartile	Maximum	Mann-Whitney U-test
Histopathological type	Borderline	3.13	2.36	0.00	2.00	2.50	4.00	8.00	H = 4.4292 *p* = 0.3510
	Clarocellulare	3.70	2.71	0.00	3.00	3.50	4.00	8.00	
	Endometrioidales	3.13	2.36	0.00	2.00	2.50	4.00	8.00	
	Mucinosum	4.00	0.00	4.00	4.00	4.00	4.00	4.00	
	Serosum	4.23	1.83	2.00	4.00	4.00	4.00	8.00	
Stage	IA	3.45	2.03	0.00	2.00	4.00	4.00	8.00	Z = 0.7566 *p* = 0.4493
	Other	4.15	2.48	0.00	3.00	4.00	4.00	8.00	
Grade	G1	4.38	1.51	3.00	4.00	4.00	4.00	8.00	Z = 1.3584 *p* = 0.1743
	G2	3.62	2.30	0.00	2.00	4.00	4.00	8.00	
Menopausal status	Postmenopausal	3.38	1.82	0.00	2.00	4.00	4.00	8.00	Z = 0.5567 *p* = 0.5777
	Premenopausal	4.31	2.78	0.00	2.00	4.00	8.00	8.00	

## Discussion

Zinc fingers (ZNFs) are one of the most abundant groups of proteins with a wide range of molecular functions. Given the wide variety of zinc finger domains, ZNFs are able to interact with DNA, RNA, PAR (poly-ADP-ribose) and other proteins. Thus, they are involved in the regulation of several processes. Recent evidence points to important role of ZNFs in initiation and progression of carcinogenesis. The zinc finger family includes both tumor suppressor genes and oncogenes [26, 27]. ZNFs are involved in all major pathways of cancer progression, from carcinogenesis to metastasis formation. Moreover, acting as transcription factors, they play a role in cancer development. Finally, a growing body of evidence suggests that zinc finger proteins may act as recruiters of chromatin modifiers or structural proteins that regulate the migration and invasion of cancer cells. Increased cellular motility is a key determinant of cancer progression, enabling migration, invasion and formation of metastases. Other processes playing a key role in the initiation and progression of ovarian malignancies may also depend, at least in part, on the interaction between different types of committed stem cells within the ovary and surrounding microenvironment [28]. Upregulation of pro-inflammatory cytokines, which is typical for ovulation, may contribute to creation of a local microenvironment which favors transformation of normal ovarian epithelial cells within the ovary; subsequently, the transformed ovarian epithelial cells may undergo immunoediting which orchestrates the interaction between infiltrating immune cells and ovarian stromal microenvironment toward EOC progression [29]. In our present study, we found the expressions of ZNF143 and ZNF 281 in 90 and 57% of examined specimens, respectively. This implies that both borderline ovarian tumors and low-grade ovarian cancers may undergo extensive processes associated with EMT initiation. Metastatic spread is a primary determinant of poor prognosis in cancer patients, and EMT plays a key role in cancer invasion and metastasis formation [30]. Based on the intensity of ZNF143 and ZNF 281 expressions in serous borderline tumors and low-grade ovarian cancers, one may hypothesize that metastasis formation and spread of these malignancies involve some additional, yet unidentified, mechanisms. Cellular changes associated with EMT co-exist with modifications at protein and gene levels, such as downregulation of epithelial intermediate filament-forming proteins (cytokeratins), overexpression of type III mesenchymal intermediate filament protein (vimentin) and alterations of cell-cell and cell-matrix adhesion molecules [31]. Another key feature of EMT is the so-called “cadherin switch”, i.e. a downregulation of epithelial cadherin (E-cadherin) with a concomitant upregulation of neural cadherin (N-cadherin) [32]. This process, postulated to enhance cell motility and invasiveness [33], seems to be regulated by a number of transcription factors that negatively modulate E-cadherin expression, among them SNAIL, SLUG, TWIST1/2 and ZEB1/2 [34, 35].

Surgical staging is an essential component of management in all women with ovarian malignancies. The staging provides vital prognostic information; in particular, it facilitates the decision whether a given patient requires an adjuvant treatment or not. In the past, surgical staging for ovarian cancer required exploratory laparotomy to perform various procedures advised by the FIGO: hysterectomy and salpingo-oophorectomy, pelvic and para-aortic lymph node dissections, omentectomy, peritoneal washings and peritoneal biopsies [36]. However, chemotherapy is given to all patients, either those with visible deposits of ovarian cancer cells within the abdomen after debulking surgery, or those without. In all patients included in our present study, ovarian tumors were removed during the exploratory laparotomy. Nowadays, however, primary debulking surgery can be carried out using minimally invasive techniques in some selected cases. Surgeons can now perform all necessary procedures required for comprehensive surgical staging using laparoscopy or robotic-assisted laparoscopy [37]. The advantages of laparoscopic surgery over laparotomy are well-established and include better intraoperative visualization, smaller incisions, reduced blood loss, lesser postoperative complications, such as wound infections and small bowel ileus, shorter hospital stay and expedited recovery [38]. However, the minimally invasive procedures may also pose some risks for ovarian cancer patients, including laparoscopic dissemination of cancer cells and higher likelihood of port site recurrence [39, 40]. Regardless the method of primary debulking surgery, accurate determination of ovarian tumor type based on its histological, immunohistochemical and molecular characteristics is of utmost importance in the context of optimizing the treatment outcomes.

ZNF143 regulates many cell cycle-associated genes, and binding sites for ZNF143 have been found in approximately 2000 mammalian promoters, which implies that this protein plays a role in a variety of cellular processes [41]. Indeed, previous studies demonstrated that ZNF143 is involved in cell cycle control, cell viability and drug resistance [14, 42]. Plausibly, this protein might not only regulate basic cellular functions at the transcriptional level but also could be involved in the control of cell proliferation, likewise the well-known proliferation marker, Ki67 (MIB-1) [13]. The DNA-binding domain of ZNF143 is located in the central part of the molecule [42–44]. The target genes of ZNF143 were shown to be fundamental for cancer progression [42]. ZNF143 was shown to be a determinant of cancer cell motility, as it represses ZEB1, which results in upregulation of E-cadherin to maintain epithelial characteristics of proliferating cancer cells in response to IGF-1. Moreover, ZNF143 is implicated in the regulation of E-cadherin expression through ZEB1, and the loss of E-cadherin by the ZNF143-ZEB1-linked cascade was shown to be related to cancer cell motility [45, 46]. The expression of ZNF143 is known to be activated by the reagents causing DNA damage, such as etoposide, cisplatin and doxorubicin [47] Moreover, ZNF143 was shown to bind to cisplatin-modified DNA and to be involved in the development of cisplatin resistance [48, 49]. In line with the conventional model of ovarian cancer progression, cancer cells develop resistance after one or multiple courses of chemotherapy, and the chemoresistance is a key determinant of cancer-related mortality [50]. From a clinical perspective, patients with EOCs usually respond well to initial surgical cytoreduction and chemotherapy, but later, most of them develop drug-resistant recurrence, probably as a consequence of ovarian cancer drug-resistant cells’ ability to evade the first line chemotherapy [51]. Despite an initially favorable response, up to 80% of women without visible evidence of the disease after the first line chemotherapy will experience recurrence and die of ovarian cancer within 12 years of diagnosis [52, 53]. According to Giannakeas et al., a small proportion of cells are chemoresistant from the outset, and chemotherapy does not contribute to the development of resistance among previously sensitive cells, but rather favors selection of the existing resistant clones. The proportion of chemoresistant cells in the ovarian cancer mass increases with the number of chemotherapy cycles, and as a result, the resistant cells constitute the dominant cell population at the time of death [50]. One biomarker of chemoresistance is a proto-oncoprotein, FOXM1, the overexpression of which has been found in many various malignancies, including EOCs. FOXM1 was shown to reduce the sensitivity of cells to anti-cancer drugs, such as cisplatin and paclitaxel, in an in vitro model, and its overexpression in tumor tissues is a predictor of cancer progression and an unfavorable prognostic factor [54]. It cannot be excluded that also a nuclear overexpression of ZNF143 protein might be a characteristic feature of chemoresistant cells in low-grade ovarian carcinomas, but this hypothesis needs to be verified in a specifically-designed study. In our present study, we observed strong nuclear expression of ZNF143 in most examined ovarian tumors, regardless their histological subtype. No statistically significant differences were found in the intensity of ZNF143 expression in borderline ovarian tumors and low-grade ovarian cancers. According to many authors, transcription factors do not constitute a good target for drug design because they form multiple complexes with other cofactors. However, ZNF143 downregulates the expression of PLK1 and AURKB kinases [14] which play a critical role in cell cycle progression and as such, constitute a promising therapeutic target. Since ZNF143 controls both DNA replication and the expressions of cell cycle regulatory molecules, it may potentially find application in cancer diagnostics and treatment [42].

ZNF281 is located at 1q32,1 chromosome and is a critical regulator of embryonic stem cell differentiation and tissue development [55]. As an EMT-inducing transcription factor, it may play a crucial role in the control of cancer cell migration, invasion and spread [56]. Direct induction of ZNF281 transcription by SOX4 was the first evidence for its potential involvement in EMT [19]. Moreover, ZNF281 was shown to directly induce the expression of SNAIL, which is vital for the ZNF281-induced EMT [18]. Furthermore, ZNF281 regulates a number of EMT effector genes (e.g. CDH-1, OCLN and CLDN-7) directly, binding to their promoters [18]. Taken altogether, this evidence suggests that ZNF281 may contribute to the loss of cell-cell contacts and determine mesenchymal phenotype of cancer cells. According to literature, the establishment and maintenance of the cellular mesenchymal phenotype may involve an extensive crosstalk between EMT-inducing transcription factors and miRNAs [57, 58]. Ectopic expression of ZNF281 was sufficient to induce EMT in colorectal cancer cell lines with epithelial features, and ZNF281was shown to be necessary for SNAIL-induced EMT. Moreover, ZNF281 is a direct target for miR-34a which mediates its repression by the p53 tumor suppressor [18]. The fact that the downregulation of ZNF281 prevented formation of lung metastases of a colorectal cancer cell line in a xenograft mouse model, suggests that the ZNF281-mediated enhancement of EMT and/or stemness might be crucial for colorectal cancer progression. Moreover, experimental downregulation of SNAIL prevented formation of metastases [59]. Another argument for cancer-promoting effects of ZNF281 is upregulation of its mRNA observed in primary colorectal and breast malignancies. Furthermore, the upregulation of ZNF281 was shown to be associated with the recurrence of colorectal cancer 3 years after removal of the primary tumor; this implies that overexpression of ZNF281 in primary tumor tissue might have a prognostic value [18]. Given that enhanced expression of ZNF281 in colorectal cancer correlates significantly with clinical stage of the malignancy, this protein might find application in oncological diagnostics, prognosis or even treatment [20]. In our present study, we found the expression of ZNF281 in more than half of examined specimens, but only a small proportion of tumors showed strong expression of this transcription factor. Moreover, we did not find statistically significant differences in the cellular expressions of ZNF281 in borderline ovarian tumors and low-grade ovarian cancers.

Our patients with sBOTs and low-grade ovarian cancers did not differ significantly in terms of zinc finger transcription factor expressions, which implies that the expressions of ZNF143 and ZNF281 in these two types of ovarian tumors might be similar. With no doubt, a primary limitation of our study was a relatively small sample size, especially, the small number of patients with serous borderline tumors. Nevertheless, it should be emphasized that the majority of ovarian tumors included in this study tested positively for ZNF143 and ZNF281. The expression patterns of ZNF143 and ZNF281 identified in this study suggest that both sBOTs and low-grade ovarian cancers might undergo a dynamic epithelial-mesenchymal interconversion. We did not find statistically significant associations between the expressions of the analyzed transcription factors and clinicopathological characteristics of ovarian tumors, but this might be a consequence of a relatively small size of the study groups. The lack of statistically significant differences in the expressions of the zinc finger proteins in sBOTs and low-grade ovarian cancers might constitute an additional proof for common origin of these two tumor types, but this hypothesis needs to be verified in a larger group of patients.

To summarize, serum borderline tumors and low-grade ovarian cancers are diagnosed markedly less often than type 2 ovarian malignancies, in particular, high-grade serous ovarian carcinomas. Our research centered around the group of ovarian tumors which have better prognosis, are seemingly easier to treat, and in some selected cases can be even handled with minimally invasive surgical techniques. Despite this, they still deserve researchers’ attention, inter alia due to problems in the treatment of advanced and recurrent lesions, and their resistance to platinum-based chemotherapy regimens.

## Abbreviations

E-cadherin: Epithelial cadherin
EMT: Epithelial-mesenchymal transition
EOC: Epithelial ovarian cancer
EQUATOR: Enhancing the Quality and Transparency of Health Research
FFPE: Formalin-Fixed, Paraffin-Embedded
LGSC: Low-grade serous carcinoma
N-cadherin: Neural cadherin
sBOT: Serous borderline ovarian tumors
STAF: Selenocysteine tRNA gene transcription activating factor
STROBE: Strengthening the Reporting of Observational Studies in Epidemiology
ZNF143: Zinc finger protein 143
ZNF281: Zinc finger protein 281
